# Deep Convolutional Neural Network Based on Computed Tomography Images for the Preoperative Diagnosis of Occult Peritoneal Metastasis in Advanced Gastric Cancer

**DOI:** 10.3389/fonc.2020.601869

**Published:** 2020-11-02

**Authors:** Zixing Huang, Dan Liu, Xinzu Chen, Du He, Pengxin Yu, Baiyun Liu, Bing Wu, Jiankun Hu, Bin Song

**Affiliations:** ^1^ Department of Radiology, West China Hospital, Sichuan University, Chengdu, China; ^2^ State Key Laboratory of Biotherapy, Department of Gastrointestinal Surgery and Laboratory of Gastric Cancer, Collaborative Innovation Center for Biotherapy, West China Hospital, Sichuan University, Chengdu, China; ^3^ Department of Pathology, West China Hospital, Sichuan University, Chengdu, China; ^4^ Institute of Advanced Research, Infervision, Beijing, China

**Keywords:** stomach neoplasms, peritoneal neoplasms, deep learning, tomography, x-ray computed, neural networks, computer

## Abstract

We aimed to develop a deep convolutional neural network (DCNN) model based on computed tomography (CT) images for the preoperative diagnosis of occult peritoneal metastasis (OPM) in advanced gastric cancer (AGC). A total of 544 patients with AGC were retrospectively enrolled. Seventy-nine patients were confirmed with OPM during surgery or laparoscopy. CT images collected during the initial visit were randomly split into a training cohort and a testing cohort for DCNN model development and performance evaluation, respectively. A conventional clinical model using multivariable logistic regression was also developed to estimate the pretest probability of OPM in patients with gastric cancer. The DCNN model showed an AUC of 0.900 (95% CI: 0.851–0.953), outperforming the conventional clinical model (AUC = 0.670, 95% CI: 0.615–0.739; p < 0.001). The proposed DCNN model demonstrated the diagnostic detection of occult PM, with a sensitivity of 81.0% and specificity of 87.5% using the cutoff value according to the Youden index. Our study shows that the proposed deep learning algorithm, developed with CT images, may be used as an effective tool to preoperatively diagnose OPM in AGC.

## Introduction

According to the GLOBOCAN 2018 data, gastric cancer (GC) remains the fifth most common cancer and the third most deadly cancer worldwide (1). Peritoneal metastasis (PM) occurs in ~53–66% of patients diagnosed with metastatic GC (2), especially in younger patients with advanced gastric cancer (AGC) (3). Patients with PM thus may be subject to late detection or even improper surgical treatment. Therefore, the early detection and diagnosis of PM in GC patients prior to surgery would be crucial for avoiding unnecessary resection and allow for optimal therapy selection in clinical practice (4–9).

Abdominal enhanced CT is considered the most common noninvasive modality of preoperative diagnosis in GC patients (5, 7, 9–11). Typical PM indications on CT images include omentum cake, extensive ascites, and parietal peritoneal thickening (12). Clinically, occult peritoneal metastasis (OPM) often refers to PM negativity on initial CT diagnosis that is revised to PM positivity following subsequent laparoscopy or surgery (12, 13). Due to the nature of OPM, it is often missed by radiologists when interpreting CT images alone, resulting in low detection sensitivity and diagnostic accuracy in AGC patients. It has been reported that approximately 16% of OPMs are missed on CT images (12, 14–16), even with multidisciplinary discussion (12, 13). MRI and PET/CT are considered second choices because they are less sensitive than abdominal enhanced CT in detecting peritoneal metastases (17–19). In addition, the costs of MRI and especially PET/CT are high. Recent advances in technology using laparoscopy have provided reliable preoperative methods to identify OPM in patients with AGC (5, 8–10, 20, 21). However, there are many medical concerns and adverse medical care issues due to its invasive and costly nature, and its application in appropriate patient selection remains controversial. Therefore, the development of a noninvasive method to facilitate the targeted diagnosis of OPM beyond conventional imaging is urgently needed.

Artificial intelligence (AI) technology, particularly deep learning, has shown remarkable progress in medical image interpretation (22–24). A typical deep learning approach, named convolutional neural network (CNN), is a novel and powerful tool for the image-based determination of complex relationships and has exhibited sophisticated performance for small feature detection and characterization (25–27). The literature has reported the use of CNNs in the detection and diagnosis of tumor diseases, such as prostate cancer, breast cancer, and lung cancer (28, 29), highlighting the value of deep learning in clinical practice.

We therefore aimed to develop a deep CNN (DCNN)-based model for the preoperative diagnosis of OPM in AGC patients and to compare its diagnostic performance with that of the conventional clinical model using logistic regression.

## Materials and Methods

This retrospective study was approved by the Biomedical Research Ethics Committee of West China Hospital of Sichuan University, and the requirement for informed consent was waived.

### Patients

**Figure 1:**
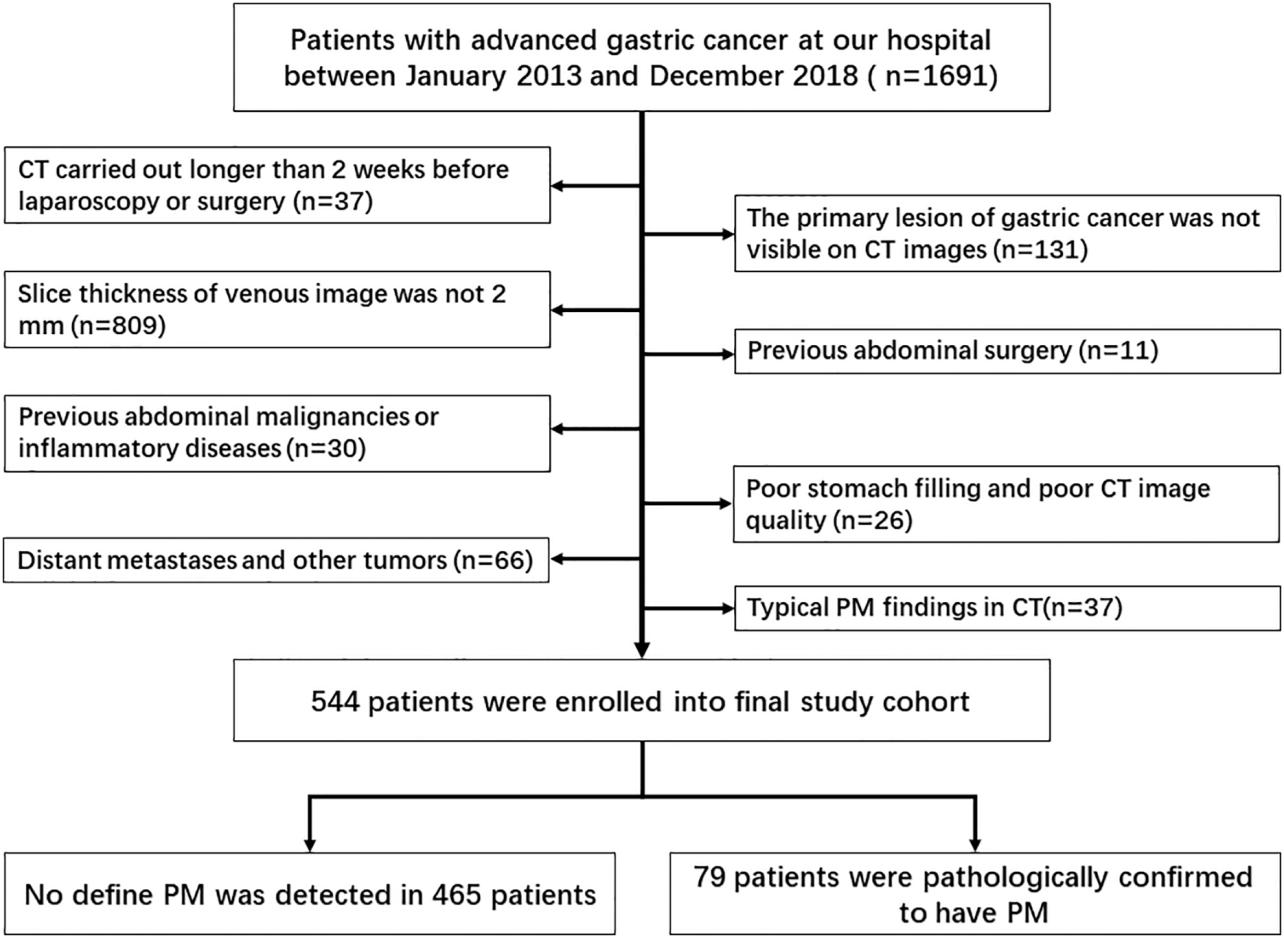
Flow chart of the selection process for the included studies.

The study was carried out at Surgical Gastric Cancer Patient Registry of West China Hospital (id: WCH-SGCPR-2019-08). Patients were enrolled based on the following inclusion criteria: (1) patients with AGC (cT ≥ 2) diagnosed by endoscopy–biopsy and CT; (2) patients who received whole abdominal enhanced CT scan preoperatively with a venous-image slice thickness of 2 mm; (3) patients without typical PM findings on CT, such as omental nodules or omental cake, extensive ascites, or irregular thickening with high peritoneal enhancement; and (4) patients with no other evidence of distant metastasis or other tumors. The exclusion criteria were as follows: (1) previous abdominal surgery; (2) previous abdominal malignancies or inflammatory diseases; (3) CT carried out more than 2 weeks before surgery; (4) poor stomach filling; (5) poor CT image quality due to artifacts; and (6) inability to visualize the primary lesion of gastric cancer on CT images. The selection process of patients included in this study is shown in **Figure 1**.

PM status confirmed: All patients were confirmed to have peritoneal metastasis by surgical or laparoscopic exploration. The laparoscopy procedure used here was a “Four-Step Procedure” of laparoscopic exploration for GC (30). During the procedure, the abdominal and peritoneal conditions were carefully examined. All suspicious peritoneal implants or ascites were sent for pathological biopsy or cytological examination. Existence of PM was determined using the American Joint Committee on Cancer guidelines in consensus between pathologists and surgeons.

### CT Image Acquisition

Prior to CT examination, patients were requested to fast for at least 6 h and orally ingested 600–1000 mL water. Patients were first trained to hold their breath during scanning with the scan range covering the entire abdomen and then scanned using a 128-slice scanner (SOMATOM Definition AS+, Siemens Healthcare, Forchheim, Germany) and a dual-source CT system (Somatom Definition Flash, Siemens Healthcare, Forchheim, Germany) with the following parameters: tube voltage, 120 kV; amperage, 210 mAs; slice thickness, 2 mm; slice interval, 2 mm; field of view, 35–50 cm; matrix, 512 × 512; rotation time, 0.5 s; and pitch 1.0. With a trigger threshold of the aorta reaching 170 HU, a three-phase scan was obtained in the precontrast phase, the arterial phase at the trigger, and the portal vein phase 30 s after the trigger. Following an unenhanced scan, 1.2–1.5 mL/kg iodinated contrast agent [Iopamiro (370 mg I/mL), Shanghai Bracco Sine Pharmaceutical Corp Ltd, Shanghai, China] was injected intravenously at a flow rate of 2.5–3.0 mL/s using a high-pressure syringe (Medrad Stellant CT Injector System, Medrad Inc. Inianola, USA).

### Data Preparation

**Figure 2:**
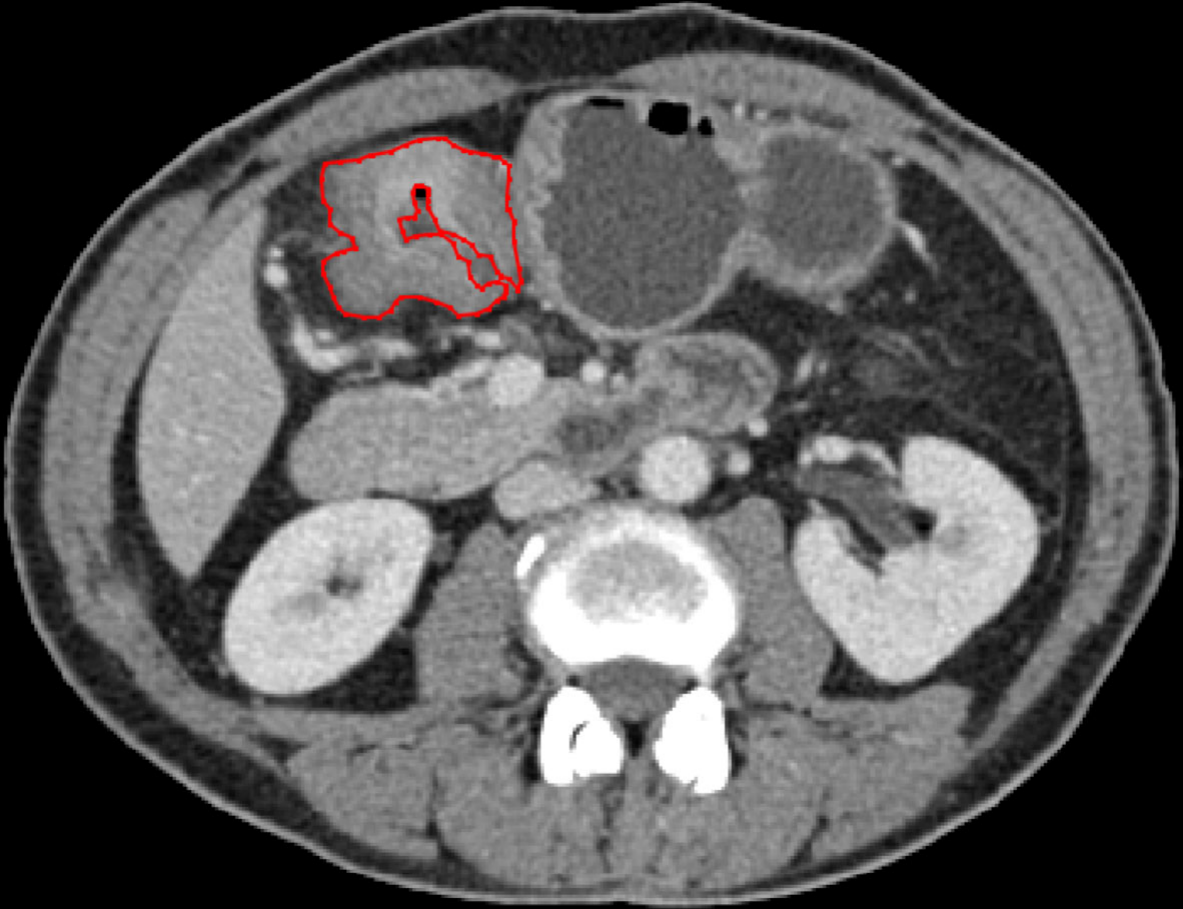
ROI annotation (red line) on a representative CT image, with the largest area of the primary lesion drawn on the axial plane.

Portal vein-phase CT images were first exported to ITK-SNAP software (version 2.2.0; www.itksnap.org) for manual segmentation. Gastric cancer lesions were then manually annotated by a radiologist (with 5 years of experience in gastroenterology imaging) and confirmed by another abdominal specialist (with 14 years of experience in gastroenterology imaging). Two radiologists reviewed all slices obtained from each patient, selected one slice with the largest tumor area and manually delineated the lesion to obtain the final regions of interest (ROIs) (**Figure 2**). The gastric lumen and artifacts were carefully avoided.

### Image Preprocessing

We first converted the images into grayscale Joint Photographic Experts Group format based on each patient’s CT images and the corresponding ROI masks. Before training the DCNN model, we applied data augmentation techniques to create new pseudoimages to expand the training sample size and enhance the model generalizability (31). Details of the data augmentation are explained in the **Supplementary Material**.

### DCNN Model

**Figure 3:**
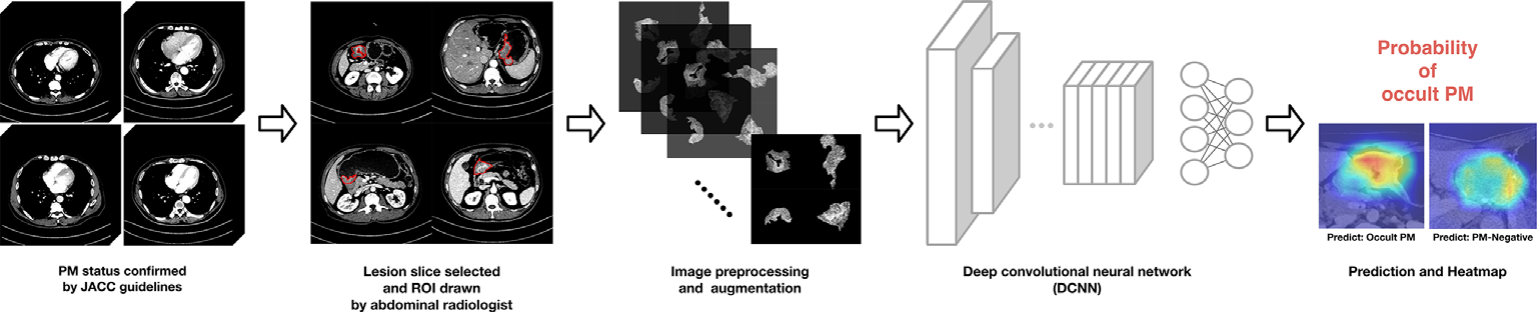
Proposed deep convolutional neural network (DCNN) workflow for OPM detection and prediction.

A simple workflow scheme for the development of the DCNN model is shown in **Figure 3**. The backbone of the DCNN model employs Xception (32), which had been pretrained on the ImageNet database (33, 34). The main structure contains a Depthwise Convolution block and a Depthwise Separable Convolution designed within the block. Considering the class imbalance among the number of PM-positive cases, we utilized a stacking strategy to train the model (35). Model training details and the stacking strategy are presented in the **Supplementary Material**.

After the training was completed, the testing cohort was used as the input into the DCNN model, and the model performance was evaluated using receiver operating characteristic (ROC) analysis. Heatmaps generated by gradient-weighted class activation mapping (Grad-CAM) were applied to display activated areas of the presence of OPM predicted by the DCNN model.

### Clinical Model

The preoperative characteristic features were applied to a multivariable logistic regression analysis to determine independent predictors of OPM. Backward stepwise selection was utilized based on the Akaike information criterion (AIC) (36). The clinical model was then developed based on the independent characteristic features and applied to the testing cohort. The diagnostic performance of the clinical model was assessed using ROC analysis. The cutoff for the ROC curve was determined by the Youden index.

### Statistical Analysis

Considering the class imbalance in the testing cohort, we used bootstrapping (n = 1000) to calculate the 95% confidence intervals (CIs). A decision curve was plotted to evaluate model efficacy by quantifying the net benefits at different probability thresholds. Differences in continuous variables were analyzed with the independent t-test, and differences in categorical variables were analyzed with the chi-squared test. DeLong’s test was used to compare the ROC curves of models. The generalizability of the DCNN model was evaluated in subgroups of age and sex using ROC curves. All statistical analyses were performed with R software (version 3.5.0; http://www.Rproject.org) and SPSS 22.0 (IBM, Armonk, NY, USA). A two-tailed *p* value lower than 0.05 was considered statistically significant.

## Results

Finally, a total of 544 patients (age, 17–87 years; median age, 60 years), including 359 men and 185 women, were enrolled in this study. All patients were confirmed with advanced GC based on end-point diagnoses, among who 79 patients were confirmed as OPM-positive using laparoscopy, while the rest (n = 465) were defined as PM-negative. **Table 1** shows the enrolled patients’ demographic information. The determination of OPM presence is described in detail in the **Supplementary Material**. None of the patients were treated with neoadjuvant chemotherapy.

**Table 1:** Characteristics of patients in the training and testing cohorts.

Characteristic	Training cohort (N = 395)				*p*	Validation cohort (N = 149)				*p*
	OPM Pos(N = 58)		OPM Neg(N = 337)			OPM Pos(N = 21)		OPM Neg(N = 128)		
	Mean	Std	Mean	Std		Mean	Std	Mean	Std	
Age (years)	58.17	13.24	57.99	11.74	0.451	55.86	16.33	60.41	10.76	0.121
	N	%	N	%		N	%	N	%	
Gender					0.270					0.469
Male	34	58.6	226	67.1		12	57.1	87	68.0	
Female	24	41.4	111	32.9		9	42.9	41	32.0	
Location					0.002					0.116
U/U+M	12	20.7	68	20.2		3	14.3	36	28.1	
M/M+L	20	34.5	63	18.7		6	28.6	18	14.1	
L/L+D	18	31.0	181	53.7		9	42.9	63	49.2	
U+E	1	1.7	11	3.3		0	0.0	5	3.9	
Whole stomach	7	12.1	14	4.2		3	14.3	6	4.7	
Borrmann type					0.000					0.009
Type 1, 2	48	82.8	194	57.6		18	85.7	71	55.5	
Type 3, 4	10	17.2	143	42.4		3	14.3	57	44.5	
CEA					0.313					0.128
normal	36	62.1	235	69.7		18	85.7	88	68.8	
elevated	22	37.9	102	30.3		3	14.3	40	31.2	
CA19-9					0.091					0.221
normal	37	63.8	254	75.4		11	52.4	88	68.8	
elevated	21	36.2	83	24.6		10	47.6	40	31.2	

p was derived from the univariable association analyses between each characteristic and PM status. peritoneal metastasis, PM; Positive, POS; Negative, Neg; Standard deviation, Std; number, N; Upper stomach, U; Middle stomach, M; Lower stomach, L; Duodenum, D; Esophagus, E; carcinoembryonic antigen, CEA; carbohydrate antigen 19-9, CA19-9.

The entire cohort of 544 patients was randomly divided into a training set comprising 395 patients (58 OPM-positive and 337 PM-negative) and a testing set comprising 149 patients (21 OPM-positive and 128 PM-negative).

### Clinical Characteristics in the Training and Testing Cohorts

As shown in **Table 1**, there were significant differences between lesion location and Borrmann type for the OPM-positive and PM-negative groups in the training cohort (*p* < 0.001). There was no significant difference in age, sex, carcinoembryonic antigen (CEA), or carbohydrate antigen 19-9 (CA19-9) between the OPM-positive and PM-negative groups in the entire cohort.

### Diagnostic Performance Measurements

#### Clinical Model

As shown in **Table 2**, multivariable logistic regression analysis identified the Location-L/L+D and Borrmann type as independent predictors (*p* < 0.05) for OPM positivity and PM negativity. A clinical model that incorporated the independent predictors was developed, and a ROC curve was created. The area under the ROC curve (AUC) was 0.670 (95% CI: 0.615–0.739). The sensitivity of the clinical model for the testing cohort was 85.7%, with a specificity of 44.5% (**Table 3**).

**Table 2:** Variables and coefficients of the clinical model.

Variable	Clinic model		
	β	OR (95% CI)	*p*
Intercept	-2.2351	–	–
L/L+D	-0.7910	0.453(0.247–0.832)	0.010
Borrmann type	1.1324	3.103(1.504–6.401)	0.002

OR, Odds Ratio; CI, Confidence interval.

**Table 3:** Model performance and DeLong’s test.

Performance	Testing cohort	
	DCNN model	Clinical model
TP	17	18
TN	112	57
FN	4	3
FP	16	71
Sensitivity	0.810	0.857
Specificity	0.875	0.445
AUC	0.900 (0.851–0.953)	0.670 (0.615–0.739)
*p*	\	< 0.001

DCNN, deep convolutional neural network; TP, True Positive; TN, True Negative; FN, False Negative; FP, False Positive; AUC, area under curve.

#### DCNN Model

**Figure 4:**
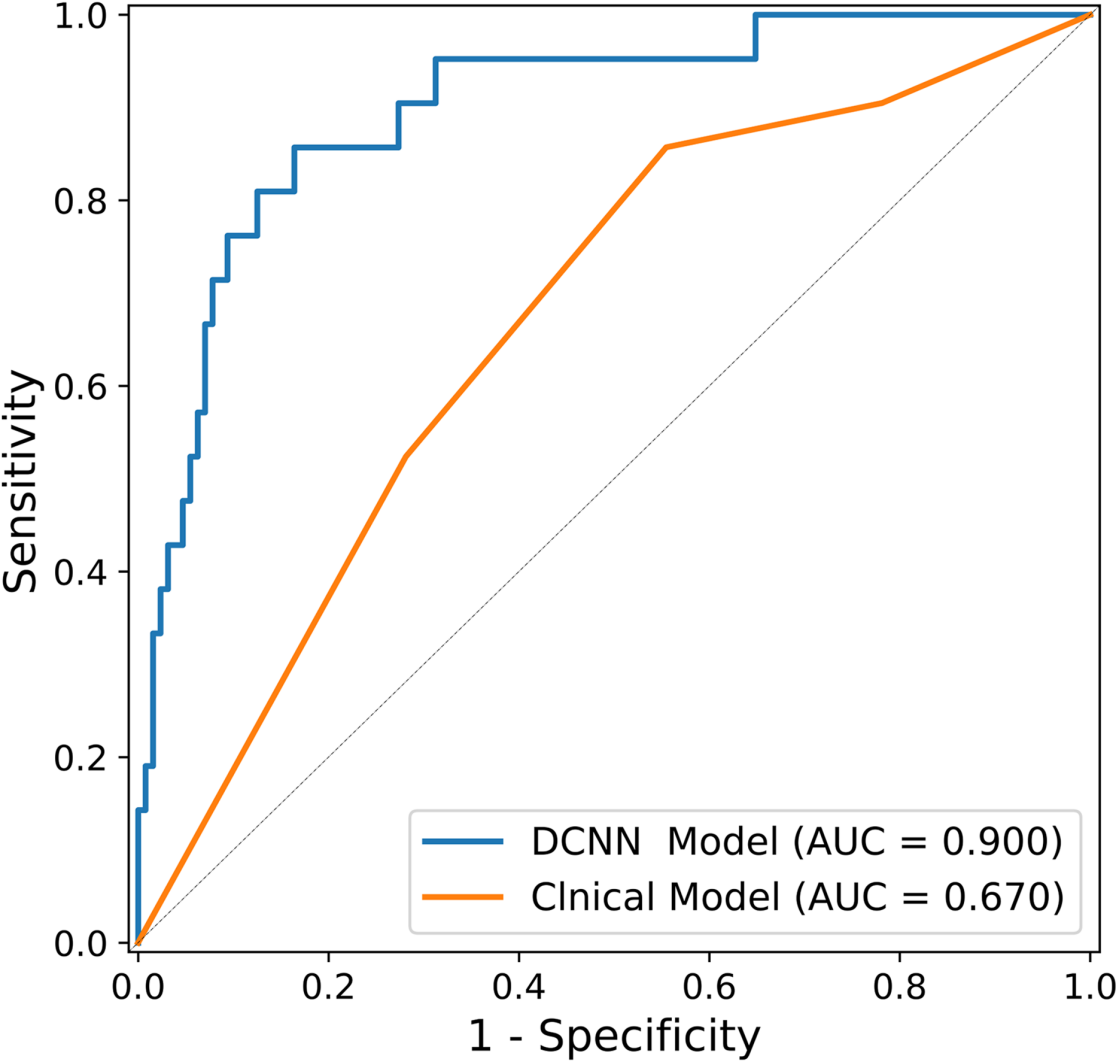
ROC curves of the DCNN model and the clinical model on the testing dataset (n = 149).

Along with the performance generated in the clinical model, **Table 3** also shows the diagnostic performance of the DCNN model using the same testing cohort. In the bootstrapping validation, the DCNN model yielded an AUC of 0.900 (95% CI: 0.851–0.953), a sensitivity of 81.0% and a specificity of 87.5%. Comparison of the ROC curves of the models suggested that the DCNN model significantly outperformed the clinical model (p<0.001). The ROC curves of the DCNN model and clinical model are shown in **Figure 4**.

### Stratified Analysis of Sex and Age

Deep learning algorithms frequently suffer from issues of generalizability. To test the generalization ability of the proposed DCNN model, we performed stratification analysis on the testing set subgrouped by sex and age. As shown in **Table 4**, the DCNN model presented good accuracy in the discrimination of OPM positivity and PM negativity among the different subgroups.

**Table 4:** Stratified analysis for DCNN model in the testing cohort.

Performance	Age		Gender	
	< 60	≥60	Female	Male
Sensitivity	0.800	1.000	0.889	0.833
Specificity	0.980	0.718	0.902	0.862
AUC	0.896	0.904	0.924	0.888
95% CI	0.811–1.000	0.845–0.955	0.851–0.989	0.825–0.956

DCNN, convolutional neural network, AUC, area under curve, CI, Confidence interval.

### Heatmap Analysis

**Figure 5:**
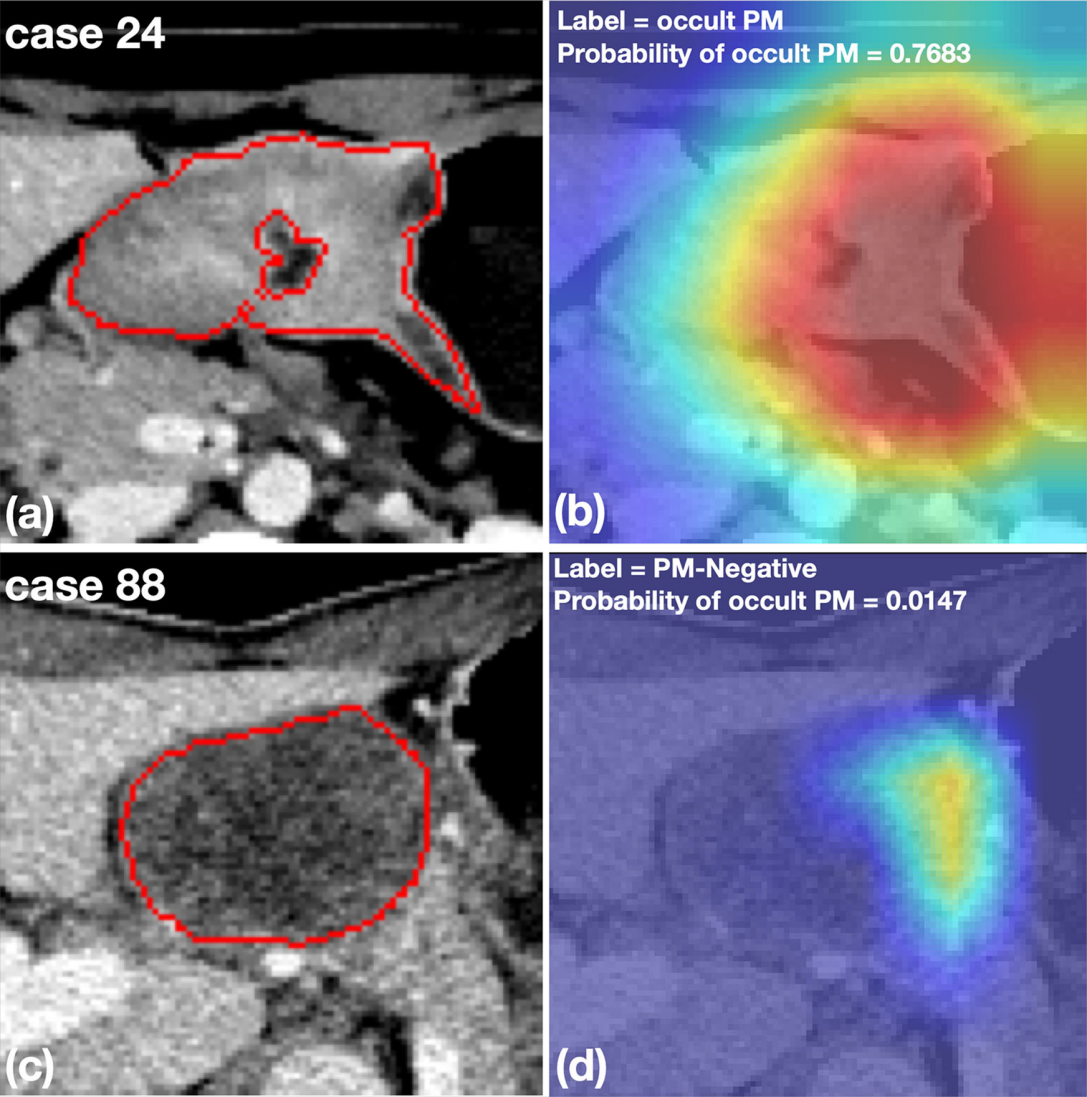
Representative cases with cropped CT images and heatmaps generated by Grad-CAM. **(A, B)** An OPM-positive patient with pathologically confirmed peritoneal tumor implants during surgery. The DCNN model correctly diagnosed the OPM region with the highest probability of 0.7683. **(C, D)** A PM-negative patient who was misclassified by the DCNN model with a probability of OPM of 0.0147. The subsequent surgery confirmed the patient to have no PM in the peritoneum.

**Figure 5** shows two representative cases with a cropped Grad-CAM view superimposed heatmaps on the original CT images. The OPM-positive case 24 was diagnosed by the DCNN model with a prediction score of 0.768, exhibiting activated status in the highlighting subregions. Furthermore, PM-negative case 88 was diagnosed by the DCNN model with a prediction score of 0.015, exhibiting inactivated status in all subregions.

### Clinical Use

**Figure 6:**
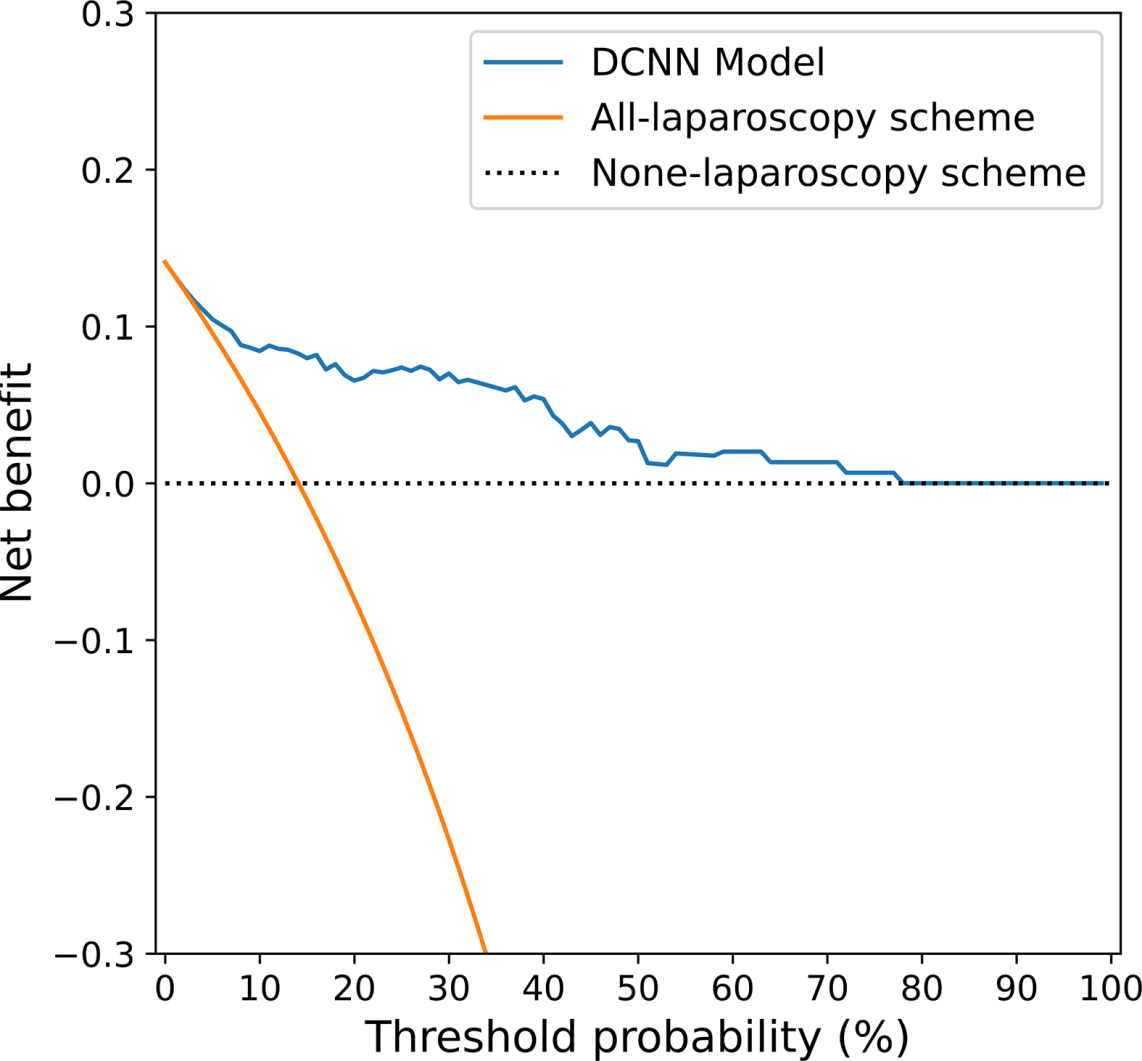
Decision curve analysis for the DCNN model and the all-laparoscopy and no-laparoscopy schemes. Blue line: DCNN prediction model, where the probability of predicting OPM ranges from a minimum of 0 to a maximum of 80%; orange line: all-laparoscopy scheme, assuming all patients should undergo laparoscopy to confirm the presence of OPM; dotted line: no-laparoscopy scheme, assuming no possibility of PM in patients (i.e., the presence of OPM).

The decision curve shown in **Figure 6** was used to compare the benefit of the DCNN model, all-laparoscopy and no-laparoscopy schemes. We found that if the threshold probability for the clinical decision was less than 80% (i.e., if the improper surgical procedure for OPM-positive patients was considered more harmful than laparoscopic exploration), the patient would benefit more from the findings of the DCNN model than either the all-laparoscopy or no-laparoscopy schemes.

## Discussion

In this study, we developed a DCNN model to identify OPM in AGC patients prior to surgical treatment. The DCNN model yielded an AUC of 0.900 and sensitivity of 81.0%, which was significantly greater than that of the clinical model (AUC of 0.532, p < 0.001). The proposed DCNN model was based on 2D images, focusing on the clinical characteristics of the primary AGC patients identified with OPM. To our knowledge, this is the first deep learning study for OPM detection and diagnosis in AGC patients.

Few published papers have focused on the preoperative assessment of PM status in GC patients (12, 14–16, 37). Previously, CT examination was chosen as the preferable diagnostic imaging modality for PM detection, while MRI and PET/CT were considered secondary choices (38). However, the reported detectability of PM on CT images varied substantially, with an average poor sensitivity of ~ 50% (13, 19). More recently, Dong et al. reported a radiomics study using CT phenotypes of primary tumors and nearby peritoneum to accurately predict OPM in AGC patients (39). While the concept of “seed and soil”, a classic theory of tumor metastasis (40), was applied in Dong’s study, the delineated ROI of the peritoneum may not have been representative of the entire “soil” condition. In our study, we focused on the characteristic features of primary tumors and their correlation with a high possibility of OPM to develop a predictive model powered by a DCNN.

Previous studies have shown that clinical factors, including Lauren type, Borrmann type, tumor location and differentiation degree, could be important predictors for PM (39, 41). However, preoperative biopsy findings do not typically include the Lauren type and the differentiation degree of GC; these are more often identified in postoperative pathological diagnosis. Therefore, Lauren type and differentiation degree were not included in our study. By incorporating the tumor location and Borrmann type (the independent predictors) in our clinical model, we found that the model had decreased diagnostic accuracy and an AUC of 0.670, suggesting that the involvement of only preoperative clinical features may not be effective for PM prediction in AGC patients. Compared to the clinical model, the proposed DCNN model yielded satisfactory performance and exhibited good generalization ability among patients of different ages and sexes (**Table 4**). Furthermore, the availability of heatmaps (**Figure 5**) provided a visual display of the PM detection estimated by the DCNN model, which could make it easier for surgeons and oncologists to make clinical assessments accordingly.

The decision of whether to begin surgical treatment in patients with gastric cancer is often a dilemma because of the ambiguity of the PM condition (4). Patients without PM on preoperative CT (but PM positivity at surgery) may undergo an unnecessary surgical procedure due to inadequate preoperative imaging and interpretation. The decision curve analysis (**Figure 6**) in our study provided an assessment of the value of the DCNN model. For patients with OPM (PM-negative) on conventional CT images, the proposed DCNN model is more suitable than the all-laparoscopy scheme or no-laparoscopy scheme based on the risk of PM. Similarly, if the DCNN model suggested a high possibility of OPM, it would be relatively beneficial to perform diagnostic laparoscopy for confirmation.

Our study indeed has several limitations. First, the delineated ROIs obtained from a single slice (2D) might not be representative of the entire tumor. ROIs extracted from 2D or 3D images may have an impact on model development and optimization. 3D analysis of the entire tumor is one of our further study interests. Second, we used retrospective datasets to develop the DCNN model and examined a relatively small number of clinical factors. Other factors, such as serological tumor markers, are not initially available on CT scans and may account for any incomplete data. Third, OPM samples were enrolled in the study cohort based on the combined results from initial CT examination (negative) and laparoscopy (positive), which limited the sample size. Finally, external validation is needed to assess the model’s diagnostic performance and generalizability across different medical institutions.

In conclusion, compared to a conventional clinical model built using logistic regression, the proposed DCNN model achieved superior diagnostic accuracy for OPM detection and diagnoses in AGC patients. The DCNN model may have significant clinical implications for early detection and proper surgical treatment for patients with AGC.

## Data Availability Statement

The original contributions presented in the study are included in the article/**Supplementary Material**. Further inquiries can be directed to the corresponding authors.

## Ethics Statement

The studies involving human participants were reviewed and approved by Biomedical Research Ethics Committee of West China Hospital of Sichuan University. Written informed consent for participation was not required for this study in accordance with the national legislation and the institutional requirements.

## Author Contributions

ZH, JH, and BS conceived and designed the study. ZH drafted the original paper. DL, XC, and DH extracted all data and performed the analyses. PY and BL conducted deep learning modeling and statistical analysis based on data. BW, JH, and BS supervised the project and provided direction and guidance throughout the preparation of this manuscript. BS revised the final manuscript. JH provided funding for the study. All authors contributed to the article and approved the submitted version.

## Funding

This work was supported by the 1*3*5 Project for Disciplines of Excellence, West China Hospital, Sichuan University (no. ZY2017304).

## Conflict of Interest

The authors declare that the research was conducted in the absence of any commercial or financial relationships that could be construed as a potential conflict of interest.
