# Supplementary figures and images for: Deep Convolutional Neural Network Based on Computed Tomography Images for the Preoperative Diagnosis of Occult Peritoneal Metastasis in Advanced Gastric Cancer
Source: Front Oncol. 2020 Nov 2;10:601869. doi: 10.3389/fonc.2020.601869 (PMC7667265; doi:10.3389/fonc.2020.601869)

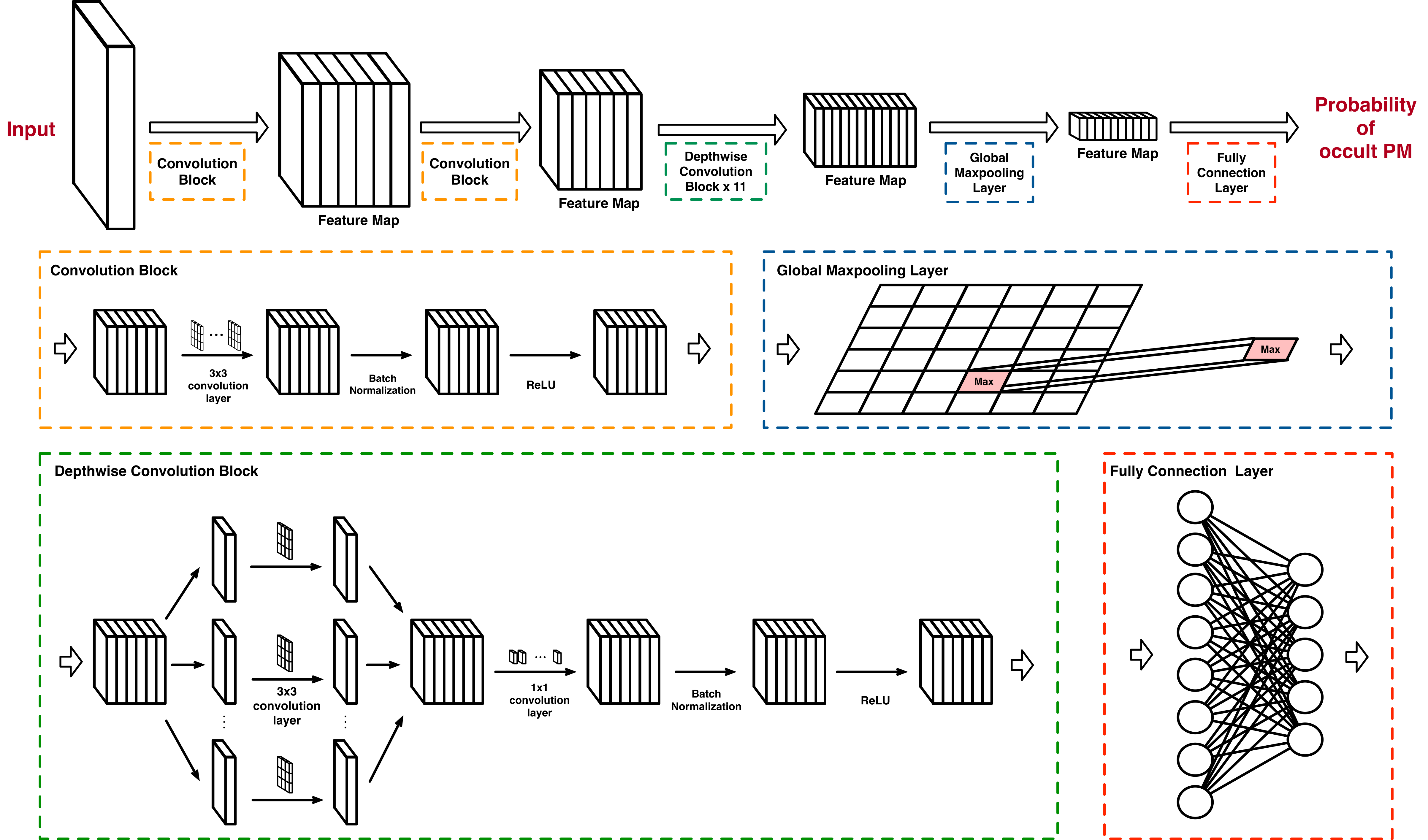

Supplement: Supplementary file 1 [file Image_1.tif]

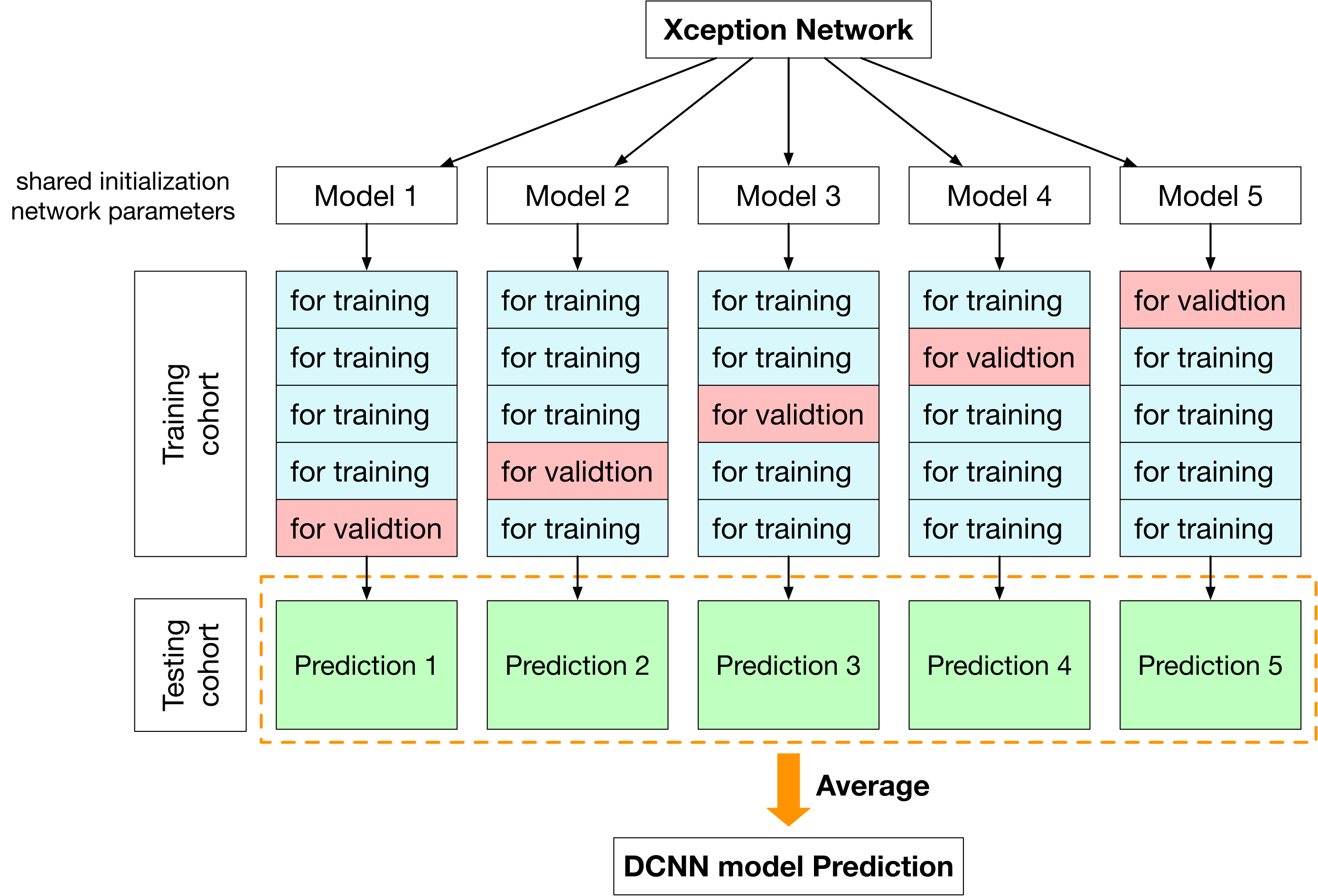

Supplement: Supplementary file 2 [file Image_2.tif]
