# Supplementary material for: Deep Convolutional Neural Network Based on Computed Tomography Images for the Preoperative Diagnosis of Occult Peritoneal Metastasis in Advanced Gastric Cancer
Source: Front Oncol. 2020 Nov 2;10:601869. doi: 10.3389/fonc.2020.601869 (PMC7667265; doi:10.3389/fonc.2020.601869)
Supplement: Supplementary file 3 [file Table_1.docx]

Supplementary Material

**Imaging preprocessing**

Two abdominal radiologists reviewed all slices of each CT scan and selected one slice with the largest tumor area and manually drawn along the margin of the lesion to obtain the region of interests (ROIs). To avoid erroneous learning noises, only delineated ROIs were used as the input data into the deep convolutional neural network (DCNN) models. Due to the unbalanced classes between occult PM-positive and PM-negative cases, the training data was amplified to balance the ratio of class labels(2). All images were rotated randomly within ± 20º to enhance model generalizability. The regions around the primary tumors were re-sized randomly by up to 40%, and images were flipped horizontally. Then, all augmented training datasets were re-sized to 96 × 96 pixels to standardize the scale, and images were subsequently entered into the DCNN model. Only Real-time data augmentation created by augmentation method was applied to the DCNN model, which allowed for dynamic control during the training phase.

**DCNN model**

The DCNN model was developed using PyTorch (available at <https://pytorch.org/>), an open-source machine learning library from the Facebook Team. Model training was implemented on a computer with 64 GB of random access memory, a Core i7-6700K 4.00-GHz central processing unit (Intel) and a GeForce GTX 1080 graphics processing unit (NVIDIA), using the Python 3.6 programming language and the Chainer 1.24.0 framework for neural networks (http://chainer.org/).

The backbone of the DCNN model employed Xception, which has been pre-trained on the ImageNet database, containing a Depthwise Convolution block and a Depthwise Separable Convolution designed within the block (**Supplementary Figure 1**) (3, 4). The batch size was set to 16 ,and the learning rate was set to 0.0003. We used the adaptive moment estimation (Adam) optimizer, with the momentum set to 0.9 and weight decay to 0.0005 in order to optimize binary cross-entropy(5). Layer dropout was applied to remove neurons from the fully connected layers to prevent overfitting at each training iteration(6). One dropout layer was set after the final Depthwise Convolution block and the dropout parameter was set to 0.2. Model weight values were created for each epoch (one iteration through the entire cohort) and trained up to 100 epochs. **Supplementary Figure 2** showed the stacking strategy in this study(7). The five models based on Xception network architecture shared initialization network parameters. All models were trained and verified using different subsets of the training cohort. Finally, five models with the same structure but different network parameters were obtained. To get the final prediction of each case in the testing cohort, the average predict value of five models was used.

**REFERENCES**

1. Liu K, Chen XZ, Zhang WH, Zhang DY, Luo Y, Yu Y, et al. "Four-Step Procedure" of laparoscopic exploration for gastric cancer in West China Hospital: a retrospective observational analysis from a high-volume institution in China. *Surg Endosc*. (2019) 33:1674-82. doi:10.1007/s00464-018-6605-2

2. Wong SC, Gatt A, Stamatescu V, McDonnell MD. Understanding data augmentation for classification: when to warp. *2016 INTERNATIONAL CONFERENCE ON DIGITAL IMAGE COMPUTING: TECHNIQUES AND*. (2016) :59-64.

3. Russakovsky O, Deng J, Su H, Krause J, Satheesh S, Ma S, et al. ImageNet Large Scale Visual Recognition Challenge. *International Journal of Computer Vision*. (2015) 115:211-52. doi:10.1007/s11263-015-0816-y

4. Chollet F. Xception: Deep Learning with Depthwise Separable Convolutions [arXiv]. *arXiv*. (2016) :14 pp.-14 pp.

5. De Boer PT, Kroese DP, Mannor S, Rubinstein RY. A tutorial on the cross-entropy method. *ANNALS OF OPERATIONS RESEARCH*. (2005) 134:19-67. doi:10.1007/s10479-005-5724-z

6. Srivastava N, Hinton G, Krizhevsky A, Sutskever I, Salakhutdinov R. Dropout: A Simple Way to Prevent Neural Networks from Overfitting. *JOURNAL OF MACHINE LEARNING RESEARCH*. (2014) 15:1929-58.

7. WOLPERT DH. STACKED GENERALIZATION. *Neural Netw*. (1992) 5:241-59. doi:10.1016/S0893-6080(05)80023-1

**Figure Legends**

Supplementary Figure 1. Xception network architecture.

Supplementary Figure 2. Stacking strategy to obtain the final DCNN model prediction.
